# The Influence of Laser Cleaning Treatment on the Quantum Efficiency of the Most Used Metallic Photocathodes: An Overview

**DOI:** 10.3390/ma18030690

**Published:** 2025-02-05

**Authors:** Alessio Perrone, Muhammad R. Aziz, Nikolaos A. Vainos

**Affiliations:** 1 Dipartimento di Matematica e Fisica “E. De Giorgi”, Università del Salento, 73100 Lecce, Italy; alessio.perrone@unisalento.it; 2 INFN—Istituto Nazionale di Fisica Nucleare, 73100 Lecce, Italy; 3 Photonics Nanotechnology Research Laboratory (PNRL), Department of Materials Science, University of Patras, 26504 Patras, Greece; vainos@upatras.gr

**Keywords:** electron gun, metallic and superconducting photocathodes, laser cleaning, bulk photocathodes, thin-film photocathodes, quantum efficiency

## Abstract

This paper presents a comprehensive investigation into the quantum efficiency (QE) of metallic photocathodes used in modern high-performance radio frequency (RF) and superconducting radio frequency (SRF) guns. The study specifically examines how laser cleaning treatment impacts the QE of these photocathodes, providing detailed insights into their performance and potential improvements for accelerator applications, and assesses the chemical and environmental factors affecting the surface composition of metallic laser-photocathodes used in modern high-performance radio frequency (RF) and superconducting radio frequency (SRF) electron guns. This paper overviews the photocathode rejuvenation effects of laser cleaning treatment. Laser cleaning removes the oxides and hydrides responsible for the deterioration of photocathodes, increases the photoelectron emission quantum efficiency (QE) and extends the operational lifetime of high-brightness electron injectors. QE enhancement is analyzed with the aim of parametric cleaning process optimization. This study excludes semiconductor and thermionic cathodes, focusing solely on the widely used bulk and thin-film photocathodes of Cu, Mg, Y, Pb and Nb. Laser cleaning enhancement of QE in Cu from 5 × 10^−5^ to 1.2 × 10^−4^, in Mg from 5.0 × 10^−4^ to 1.8 × 10^−3^, in Y from 10^−5^ to 3.3 × 10^−4^, in Pb from 3 × 10^−5^ to 8 × 10^−5^, and in Nb from 2.1 × 10^−7^ to 2.5 × 10^−5^ is demonstrated. The analysis concludes with a specialized practical guide for improving photocathode efficacy and lifetime in RF and SRF guns.

## 1. Introduction

Laser photocathodes are widely used to produce fast and high-quality electron beams. The most important parameter is the quantum efficiency (QE) for electron emission, defined as the ratio of the number of emitted electrons per photon irradiating the active surface of the cathode. Among the available photocathode types, metallic photocathodes are particularly valued for their resilience, making them highly effective under challenging operating conditions [1,2,3]. They are considered to be the best choice of electron emitters for high-brightness applications that require robust and long-lasting electron sources, including free-electron lasers (FELs) [4,5,6], energy recovery linacs (ERLs) [7,8,9] and ultrafast electron microscopy and diffraction (UEM/D) systems [10,11]. In such applications, in which a high current, high beam quality and stability are paramount, metallic photocathodes stand out due to their endurance and fast temporal response, typically faster than 50 femtoseconds, enabling superior control over electron beam generation [12,13,14]. In this work, we focus solely on metallic photocathodes, a class uniquely fitting the demands set by RF and SRF systems, and exclude semiconductor and thermionic photocathodes. The most used materials, copper (Cu), magnesium (Mg), yttrium (Y), niobium (Nb) and lead (Pb), present superior photoemissive properties, long operational lifetimes, chemical inertness, thermal emittance, low cost and compatibility with RF and SRF injector systems. However, the QE and overall operational performance of metallic photocathodes deteriorates with use, especially at high current densities, and different types of surface cleaning treatments are applied to rejuvenate their performance. However, the conventional cleaning methods, mainly mechanical, ultrasonic and chemical [15,16], can only be applied successfully to bulk metal surfaces and are inappropriate for the thin-film photocathodes that are of interest in our work. Emphasis in this work is placed on the factors of a chemical environment that are critical for photocathode longevity and high QE in RF and SRF gun systems. The metals selected are representative of a varying level of reactivity of hydrogen- and oxygen-containing molecules such as H_2_, O_2_, H_2_O, CO_2_ and hydrocarbons forming hydride and oxide surface layers [17,18]. Such surface contaminants normally degrade the QE and the operational stability. The effects can, however, be mitigated by laser cleaning, which efficiently removes the deteriorated layers [19,20,21], exposing a high-quality pure metal surface for high QE electron emission. In this context, laser cleaning impacts the surface quality and QE of metallic photocathodes.

Even though the materials and methods for the effective “stepwise laser cleaning” of metallic photocathodes have been reviewed before, our work represents a singular effort, aiming to provide deeper insights and optimum protocols. Such a methodology is particularly useful when dealing with thin-film photocathodes. By applying gradients of exposure hydration and oxidation, the layers residing on the photocathode surface are smoothly, though effectively, annihilated, and the QE becomes greatly enhanced.

This overview incorporates recent research developments and provides a comprehensive assessment of a range of laser cleaning methods, aiming to provide a useful practical guide for those seeking the highest QE and extended photocathode lifetime. Integrating chemical resilience considerations into cleaning protocols enables us to optimize the treatment and achieve maximum QE and durability. This comparative study concludes with specific guidelines for further developments of RF and SRF photocathodes for particle accelerator and free-electron laser systems, as well as other high-brightness electron source applications.

## 2. Brief on Metallic Photocathodes

Metallic laser photocathodes made of Cu, Mg, Y, Pb and Nb exhibit exceptional structural stability and long operational lifetimes. Their robustness and high reliability enable the consistent production of low-emittance electron beams over extended periods. Unlike photocathodes made of more sensitive materials, they maintain stable performance under poor vacuum conditions, even when the vacuum level fluctuates or degrades, making them ideal for long-term operation in high-brightness photoinjectors. Their facile handling, transportation and storage in ambient air environments [22,23,24,25,26,27] add further advantages, thus justifying their longevity and widespread use in highly demanding research environments [28,29,30,31,32,33,34,35,36]. A primary limitation of metallic photocathodes concerns their relatively low QE of electron emission. This drawback has been largely attributed to the high reflectivity of the metallic surface and the shallow escape depth of photoemitted electrons. Surface treatments have aimed at improving QE, even though inherent limitations are challenging to overcome. In all cases, the resulting QE levels are generally lower than those of semiconductor photocathodes [28]. Furthermore, these cathodes are susceptible to surface contamination, which can diminish QE. The chemical environment, particularly exposure to hydrogen, oxygen and carbon-based molecules, leads to the formation of hydrides and oxides, thus compromising QE over time. Even in controlled vacuum environments, residual gas molecules are continuously adsorbed onto the photocathode surface, producing gradual degradation of performance [37,38,39]. Laser cleaning treatments have emerged as a powerful solution to counteract chemical effects. They are capable of removing contaminants without introducing physical or morphological damage to the photocathode [40,41]. The process is typically applied in several stages, by adjusting the intensity and the number of laser pulses to carefully control the surface conditioning. This “stepwise laser cleaning” strategy, which can fine-tune QE to optimal levels, not only regenerates the cathode, but also restores QE after intensive usage, thus significantly extending the operational lifetime of the device [42]. This approach enables researchers to optimize the laser cleaning process for each metal type, by balancing the laser intensity, the pulse repetition frequency and the number of laser pulses per site, and achieve maximum QE while preserving surface integrity [43].

The ability to provide rapid, facile and non-invasive in situ means of rejuvenating photocathodes has made laser cleaning an indispensable technique in the maintenance and enhancement of metallic photocathodes [41,44,45,46]. These are distinct advantages over other surface treatments, which are applied ex situ and require additional equipment, materials or vacuum cycling, that may not be so effective in restoring the performance of metallic cathodes exposed to contaminants.

## 3. Chemical Environment Factors

The most used photocathode metals, Cu, Mg, Y, Pb and Nb, are addressed in this paper. They are very reactive to hydrogen- and oxygen-containing molecules such as H_2_, O_2_, H_2_O, CO_2_ and hydrocarbons. The latter are always present as residual gases in high-vacuum systems at concentrations directly related to the quality of the vacuum environment used. These gases form hydrides and oxides on the photocathode surface over time, which deteriorate the photocathode’s performance in terms of QE and operational lifetime [47,48,49,50,51,52]. Among the metals studied, Y is the most reactive mainly because of its low electronegativity. Its strong reactivity makes this material very vulnerable to oxidation and hydration processes, which lead to degradation even in high-quality vacuum conditions. On the contrary, metals such as Cu, Pb and Nb show higher resistance to oxidation and hydration, which contributes to their better operational stability even in adverse chemical environments.

In operational terms, the quality and the pressure level of the vacuum are of paramount importance, determining both the lifespan and efficacy of metallic photocathodes. Good vacuum quality in the thin-film deposition system, and in the laser cleaning and QE measurement apparatus, is always necessary to reduce the adsorption of residual gas molecules onto the cathode surface and prevent gradual degradation of the QE. Under poor vacuum conditions, the residual gas can continuously be reabsorbed onto the metal surface, react and form insulating layers, which seriously impede photoelectron emission. This process proceeds continuously over time and significantly lowers the QE and overall efficiency of RF and SRF guns.

To highlight these effects, we discuss representative cases of the chemical environment produced in vacuum reactors during gentle ablative laser cleaning. To quantify, we refer to mass spectra recorded in the vacuum chamber and the respective evolution of atomic and molecular species.

**Figure 1:**
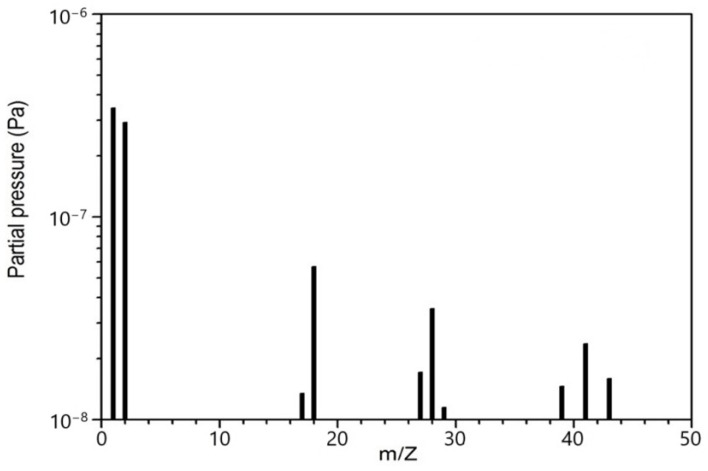
Typical time-integrated mass spectrum of residual gas at vacuum level of about 10^−6^ Pa as recorded by quadrupole mass spectrometer.

Figure 1 is a typical time-integrated mass spectrum of residual gas at a vacuum level of 10^−6^ Pa. The most intense mass peaks at 1, 2, 18 and 28 amu are, respectively, ascribed to H, H_2_, H_2_O and N_2_. The group of peaks around 40 amu is due to the electron fragmentation of acetone and iso-propyl alcohol, often used as solvents to clean UHV (~10^−7^–10^−10^ Pa) and HV (~10^−4^–10^−7^ Pa) apparatus. Molecular nitrogen is significantly less reactive than other molecules present in the residual gas; therefore, it will not be considered in this study.

**Figure 2:**
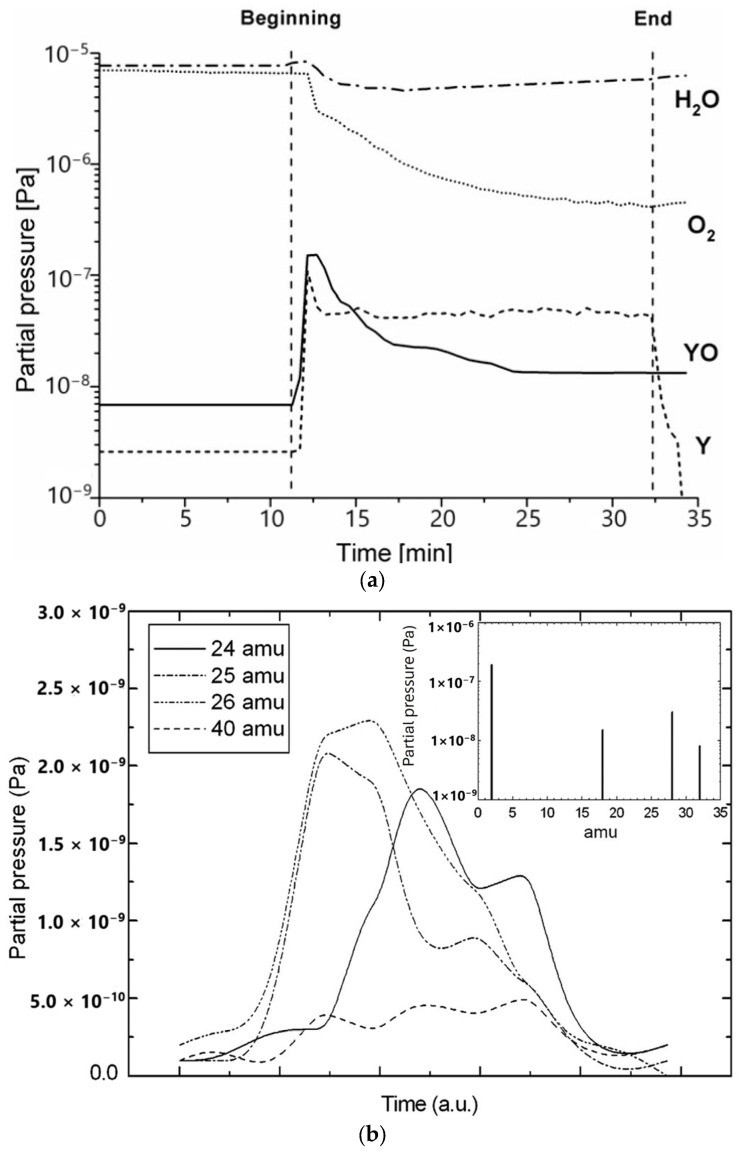
(**a**) The mass spectrum of residual gas recorded by a quadrupole mass spectrometer during the ablative laser process of a Y bulk target (reproduced from ref. [53]). The ablation process was performed by a Nd:YAG laser with a laser energy density of 8 J/cm^2^, at λ = 266 nm, τ = 7 ns and f = 10 Hz repetition rates. (**b**) The evolution of the partial pressure of the main chemical species recorded during laser ablative cleaning treatment. The inset shows the time-integrated mass spectrum before the laser cleaning procedure (reproduced from ref. [58]). For the present measurements, the 4th harmonic of the Nd:YAG laser was used to deliver pulses of 300 µJ, with a pulse duration of 30 ps and spot size of 300 µm at 266 nm.

Figure 2a presents the mass spectrum recorded during pulsed laser ablation of a cleaned Y bulk target [53]. The initial fast rise in Y partial pressure is related to the laser ablation of the Y target.

**Figure 3:**
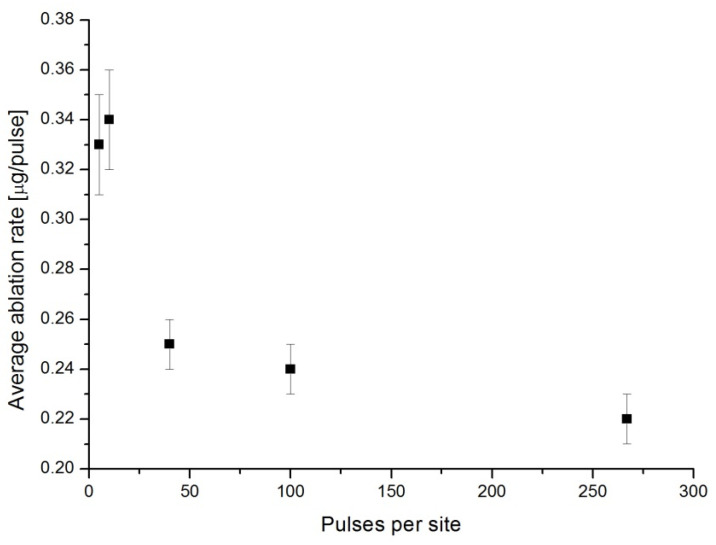
The average ablation rate of a Y target in μg/pulse as a function of laser pulses per site. The ablation process was performed by a Nd:YAG laser with a laser energy density of 8 J/cm^2^, at λ = 266 nm, τ = 7 ns and f = 10 Hz repetition rates (reproduced from ref. [53]).

The oxidation process of atoms and ions of Y present in the gas phase is confirmed by the increase in YO partial pressure. In turn, a continuous decrease in O_2_ partial pressure in the mass spectrum is observed. A similar behavior observed in the mass spectra of Y, YH, H_2_ and H_2_O is not reported here for the sake of brevity. Yet, the slight increase in H_2_O partial pressure at the beginning of the laser ablation process is related to the desorption of water vapor from the target surface. We note that after hundreds of laser pulses per site, a slow decrease in Y partial pressure is observed, which may be attributed to the drop in the average ablation rate (see Figure 3). In fact, some changes in the surface morphology [54,55,56] may be observed after repetitive irradiation even at laser energy density values lower than the nominal ablation threshold of the metal.

Figure 2b shows the mass spectrum of the chemical entities desorbed during the laser cleaning process of a Mg thin film which was deposited on a Cu substrate by pulsed laser deposition (PLD). The potential of the PLD technique to produce high-quality photocathodes based on metallic and semiconductor thin films has been reported by numerous papers [57,58,59,60]. The observed data reflect that the partial pressures of Mg (24 amu); ^25^Mg stable isotope (10% at.) and MgH (25 amu); ^26^Mg stable isotope (11% at.); MgH_2_ (26 amu); and MgO (40 amu) all increase as a function of time. The very strong peaks at 25 and 26 amu indicate that hydrides dominate the first ablated layers. The contribution of Mg isotopes to the signals at 25 and 26 amu can be ruled out, since the ablation of the underlying Mg thin film has not yet commenced. As one proceeds further into cleaning, underlying layers of the Mg thin film start becoming ablated. It is interesting to note here that the peak relating to MgO at 40 amu is weak compared to the hydride signals. This probably occurs because at a vacuum level of 10^−6^ Pa, the partial pressure of molecular oxygen is an order of magnitude lower than that of molecular hydrogen, and thus, the formation of MgO is strongly impeded, as shown in the inset of Figure 2b.

**Figure 4:**
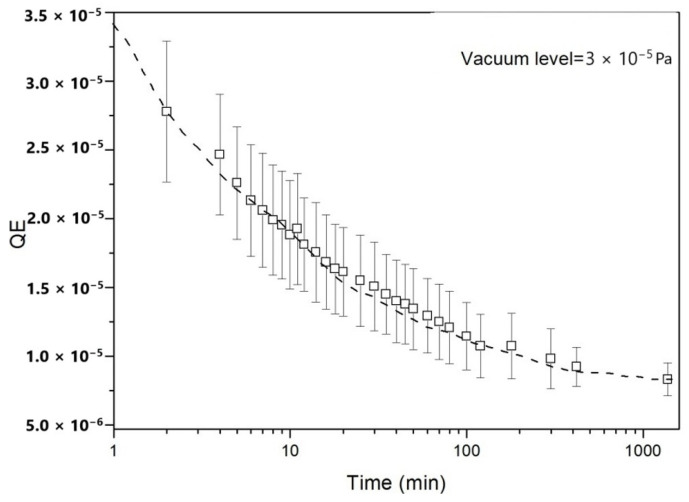
The QE of the as-deposited Y thin film by the pulsed laser deposition technique as a function of exposure time at a vacuum level of 3 × 10^−5^ Pa. The dashed line is a guide for the eye.

Figure 4 shows the QE values of the as-deposited Y thin film as a function of exposure time. The data show the strong dependence of photocathode performance on surface contamination, especially in the case of reactive metals like Y shown here. These effects can be reduced, and the useful life of metallic photocathodes can be extended, by various smart configurations that have been developed and tested. These include hybrid [61] and non-conventional configurations [62] for photocathodes based on thin films, and the friction welding [63], hot isostatic pressing [64] and press-fitting of bulk disks [65] for those based on metal bulks. The advantages and disadvantages of the different metallic photocathode configurations based on thin films are reported in ref. [66]. However, we stress that generally, such chemical degradation processes are significantly weaker for metallic photocathodes compared to semiconductor photocathodes. In addition to oxides, hydrides and other compounds formed on the surface, electron emission in semiconductors is greatly degraded, often irreversibly. Metals are generally more tolerant to surface contamination, with no drastic consequences on QE and overall performance. Highly reactive metals, like Y, require very strict vacuum conditions, and other metals, such as Cu, Pb, and Nb, exhibit good resistance and better resistivity to environmental degradation. Optimized vacuum conditions and engineered combination of materials will allow even more reliable operations over extended operational periods.

## 4. Laser Cleaning Treatment and QE Measurements

**Figure 5:**
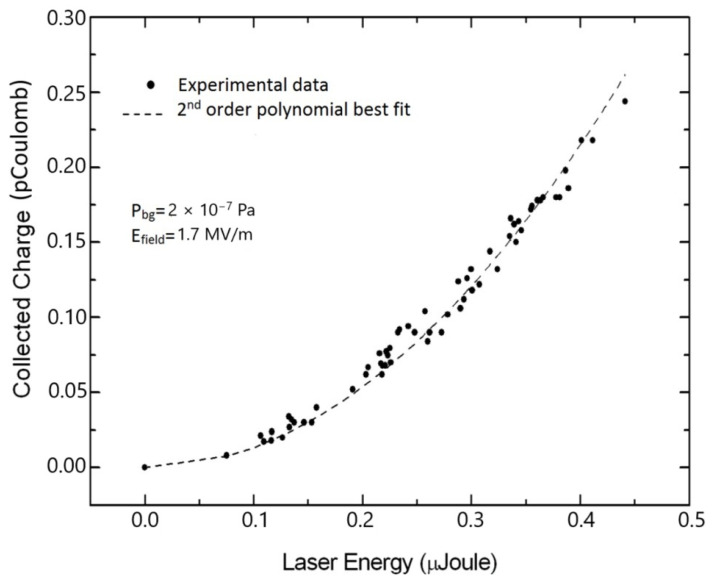
Collected charge versus laser energy for the as-deposited Mg thin film (reproduced from ref. [58]).

**Figure 6:**
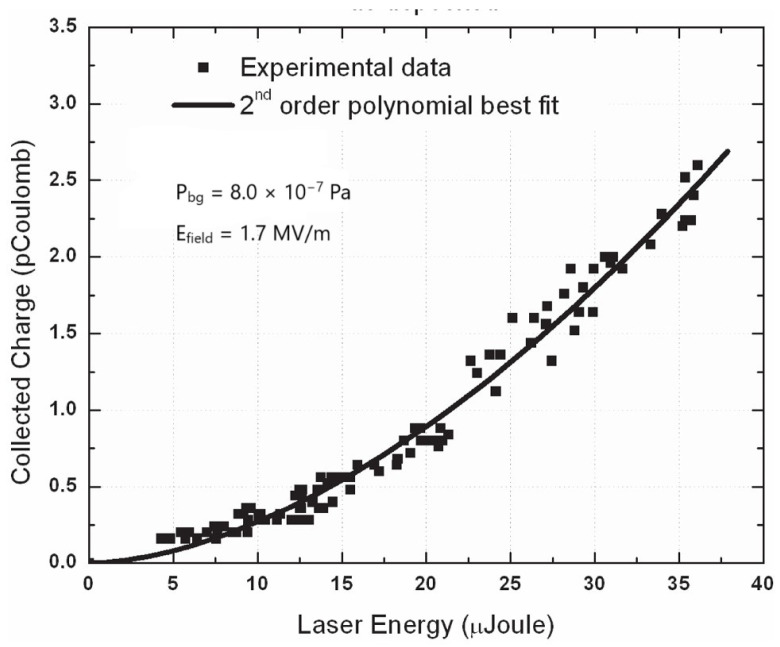
Collected charge versus laser energy for the as-deposited Y thin film (reproduced from ref. [67]).

**Figure 7:**
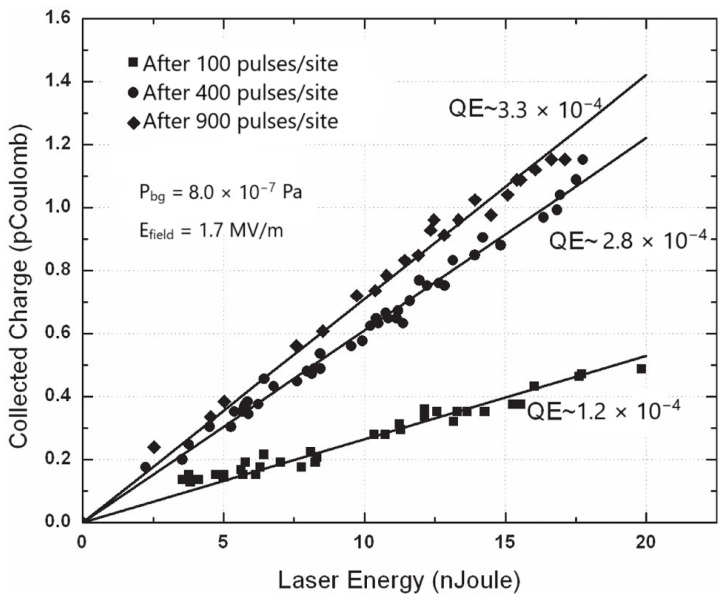
Variation in QE values after multiple laser cleaning treatments of the as-deposited Y thin film with a laser energy density of 0.04 mJ/cm^2^ (reproduced and adapted from ref. [67]).

**Figure 8:**
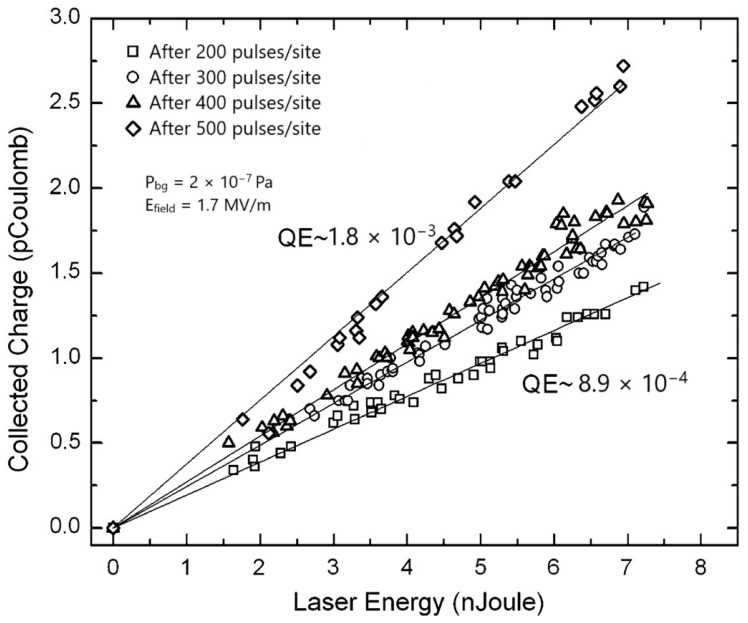
Collected charge as a function of laser energy after multiple laser cleaning treatments of the as-deposited Mg thin film with a laser energy density of 0.04 mJ/cm^2^ (reproduced and adapted from ref. [58]). As observed in the case of Y thin film, the linear relation between the collected charge and the laser energy confirms once again that the photoelectron emission process is governed by one-photon absorption. Moreover, it can be said that in the above experimental results, the space charge effect can be certainly ruled out due to the low value of a few pico-Coulombs of the collected charge.

The laser cleaning treatment of photocathodes to eliminate surface contaminants, mainly hydrides and oxides, is a typical laser ablation process. The experimentally used laser energy density on the target is set just above the laser ablation threshold in order to facilitate a gentle material removal process. Suitable energy density values are in the range of tens of mJ/cm^2^ for ps laser pulses and some tenths of J/cm^2^ for pulses in the ns regime. In any case, laser cleaning is carried out with hundreds of laser pulses per site. However, for an effective and complete cleaning process, thousands of laser pulses per site are necessary. During the measurements of QE, the laser energy density is much lower than the laser ablation threshold to avoid damage to the electron emitting surface. In all measurements reported in Figure 5, Figure 6, Figure 7 and Figure 8, similar laser parameters were used. In particular, laser pulses with a 30 ps duration and a laser spot size of about 300 μm, emitted by a mode-locked Q-switched frequency-quadrupled Nd:YAG laser (λ = 266 nm), were employed. Figure 5 and Figure 6 show the collected charge as a function of laser energy for the as-deposited Mg and Y thin films.

The quantum efficiency values were measured with a maximum error of 10% mainly due to laser intensity fluctuations. The recorded error is the result of direct measurement averaged over hundreds of pulses.

In both of the above figures, the quadratic behavior of the experimental results can be explained by the two-photon absorption process. Such photoelectric emission is due to the presence of oxides and hydrides on untreated photocathodes. The processes leading to contaminant formation have been thoroughly investigated by various research groups [68,69,70]. A protocol of consecutive in situ laser cleaning treatments was applied to eliminate the oxide and hydride layers formed on the photocathode surface. The effectiveness and importance of laser cleaning of the metallic photocathode surface is clearly shown in Figure 7 and Figure 8. In Figure 7, the measured QE values of the Y thin film after multiple laser cleaning treatments are depicted. The measured QE value increases from ~10^−5^ to 1.2 × 10^−4^ after the first cleaning treatment with 100 laser pulses per site. An increase in QE from 1.2 × 10^−4^ to 2.8 × 10^−4^ is observed after the second cleaning with 400 laser pulses per site, and finally, QE reaches its maximum at 3.3 × 10^−3^ after a total number of 900 laser pulses per site. It is important to stress here that for each laser cleaning treatment, different sites on the photocathode surface were irradiated.

The linear relationship between the collected charge and laser energy indicates that the emission process of electrons occurs mainly via one-photon absorption. Moreover, the space charge effect between emitted electrons can be ruled out. The increase in QE values with the laser cleaning procedures is closely linked to the ablation of the contaminants (oxides and hydrides) present on the photocathode surface. The chemical and physical absorption of molecules on the photocathode surface is fully explained in the previous paragraph [47,48]. A further contribution to the increase in QE after the laser cleaning treatment could also come from the reduced surface reflectivity of the cathode [54,55].

The measured QE values of the Mg thin film after multiple laser cleaning treatments are shown in Figure 8.

**Figure 9:**
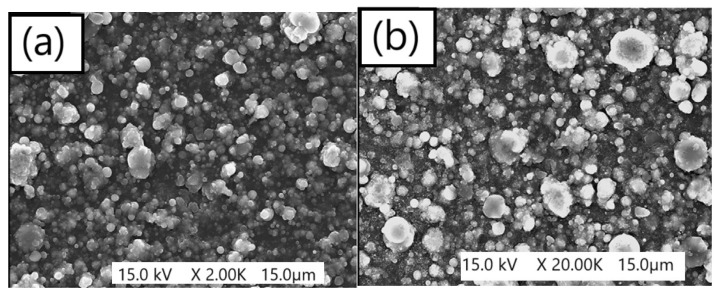
SEM micrographs of the Mg thin-film surface before (**a**) and after (**b**) the prolonged laser cleaning process with 5000 pulses per site (reproduced from ref. [59]). For the present cleaning, the 4th harmonic of the Nd:YAG laser was used to deliver pulses of 300 µJ, with a pulse duration of 30 ps and a spot size of 300 µm at 266 nm.

Figure 9 shows the SEM images of the Mg surface before and after the laser cleaning treatment.

**Figure 10:**
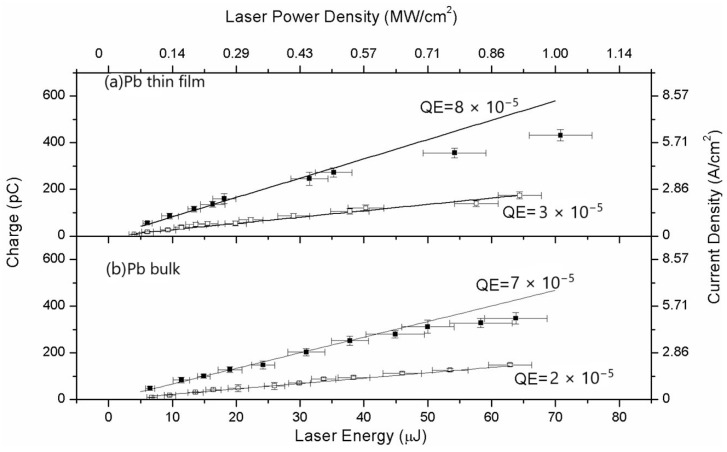
QE measurements of Pb thin film prepared by ns-PLD in hybrid configuration (**a**) and Pb bulk (**b**) before (□) and after (■) laser cleaning treatment. Cleaning was performed by Nd:YAG laser pulses at 266 nm with pulse length of 7 ns and energy density of 40 mJ/cm^2^ (reproduced from ref. [71]).

When prolonged laser cleaning with thousands of laser pulses per site is applied, the photocathode surface can be considered to be free of contaminants. Figure 10 presents the response of the Pb photocathode in thin-film and bulk form.

For clean surfaces and laser energy higher than 40 μJ, weak evidence of the space charge effect is observed. In this case, the collected charge reaches values higher than hundreds of pico-Coulombs.

**Figure 11:**
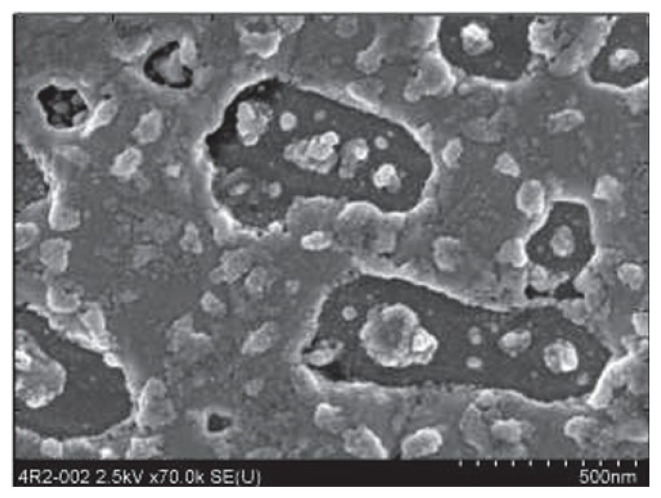
An SEM image of the Pb bulk surface before the laser cleaning procedure. Scale: 500 nm (reproduced from ref. [19]).

**Figure 12:**
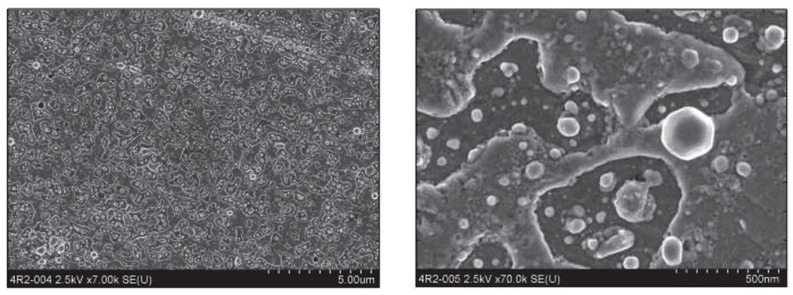
An SEM image of the Pb bulk surface after the laser cleaning procedure at two different magnifications (reproduced from ref. [19]). The cleaning was performed by 3 × 10^5^ KrF excimer laser pulses at 248 nm with a pulse length of 5 ns.

Figure 11 shows an SEM image of Pb bulk before the laser cleaning procedure, and Figure 12 just after the cleaning [19].

**Figure 13:**
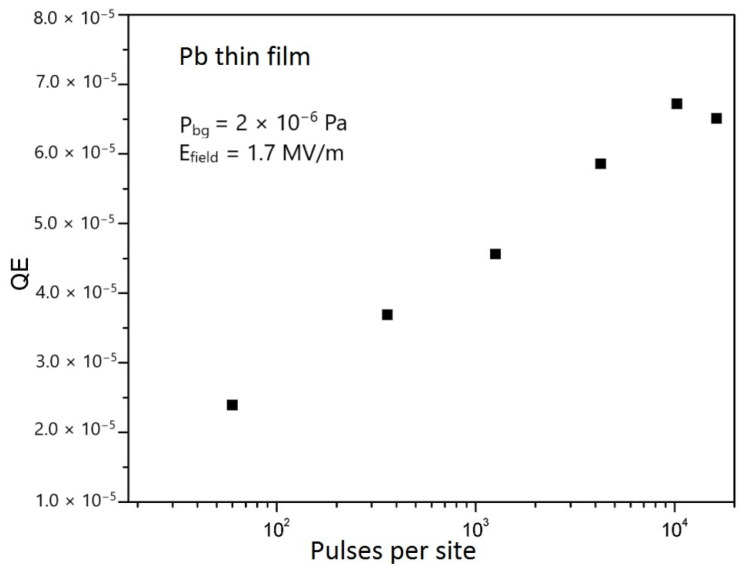
The QE of the Pb thin-film photocathode deposited with a 5 ps laser pulse duration, as a function of the total number of cleaning laser pulses per site (reproduced and adapted from ref. [72]). The cleaning was performed by KrF excimer laser pulses at 248 nm with a pulse length of 5 ps and an energy density of 0.04 J/cm^2^, and the QE measurements were carried out with a laser energy density of 0.12 mJ/cm^2^.

Figure 13 reports the QE values of a Pb thin film prepared by PLD in the ps regime as a function of the total number of cleaning laser pulses per site.

The available literature lacks articles on photocathodes built with Cu and Nb thin films. This is because there is no technological advantage to building photoemitting devices based on these two magic metals. On the contrary, a large number of articles report photocathodes of bulk Cu and Nb surfaces.

**Figure 14:**
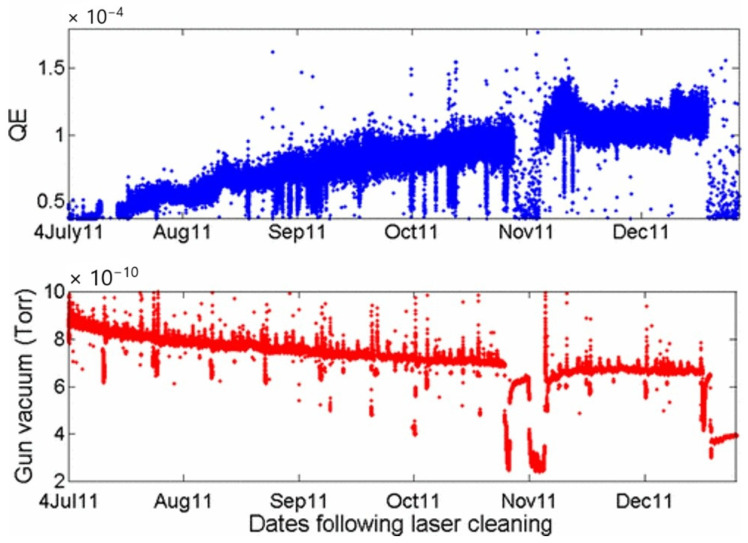
Evolution of QE and vacuum level vs. time after laser cleaning treatment (reproduced from ref. [73]).

The copper photocathode is the most used device in RF guns, mainly due to its high thermal and electrical conductivities associated with its delocalized electrons. These physical properties enhance the quality factor of RF cavities. Moreover, its chemical inertness to the aggressive gases H_2_, O_2_ and H_2_O offers a long operational lifetime of many years. Nevertheless, even photocathodes based on bulk Cu need some cleaning treatments, often carried out by focusing the photoinjector drive laser. The influence of laser cleaning treatment on the QE of bulk Cu has been studied by different research groups [73,74,75]. Figure 14 shows the evolution of QE and vacuum level vs. time for a period of 5 months after a prolonged cleaning treatment with a laser energy density of 17 μJ/cm^2^ and a laser spot size of 30 μm.

The scientific explanation of the results, shown in the above figures, is linked to the interaction of the laser radiation with the photocathode surface. It is notable in this context that the energy density of the laser was held purposely below the ablation threshold. This controlled laser pulse energy density ensures that no physical material is removed from the photocathode via ablation, which would otherwise lead to structural damage to the surface. Instead, two predominant effects are triggered by laser radiation: (1) electron photoemission, due to photons providing the electrons with the energy needed to escape the potential barrier at the metal surface, and (2) gas desorption of impurities, primarily molecular hydrogen (H_2_) and water vapor (H_2_O), which are the main chemical species inside the ultra-high-vacuum cavities [76].

In effect, the slow desorption of contaminants is the most probable reason for the continuous QE improvement with long operational time. Surface contaminants, whether originating from pre-exposure or remaining after the first laser cleaning, could block electron emission by either creating barriers or disturbing layers. However, repetitive laser irradiation seems to cause the gradual and effective removal of such surface-bound contaminants, simultaneously improving the quality of the vacuum by reducing the concentration of reactive species in the reactor vessel.

This sustainable cleaning effect is tracked by the observed gradual improvement in QE in the recorded data after prolonged operational periods, as shown in Figure 14. The surface becomes gradually more photoemissive. Furthermore, the better the vacuum quality becomes, the fewer contaminants will be available to re-adsorb, and the more QE stability and improvement there will be. Continuous contaminant removal and improved vacuum conditions not only increase QE, but also contribute to enhancing the overall operating conditions, as evidenced in Figure 14.

Photoemitting devices based on Nb bulk have also been considered. Nb resists oxidation and hydration, hence guaranteeing a high degree of chemical stability. They are appreciated as the most reliable photocathodes concerning SRF gun applications due to the high superconductivity of this metal. The superconducting state of Nb may also carry an electric current at very low temperatures with negligible resistance, a feature very important for SRF guns, which need to operate efficiently for extended periods of time at high power. This property is very vital under extreme conditions where, for instance, reactive gases or moisture could lead to the deterioration of other materials. It is under these conditions that the stability of Nb can maintain structural and chemical integrity and achieve longer operational lifetimes with less maintenance intervention. Bulk niobium photocathodes can therefore perform the best in applications of high-brightness photoinjectors, where the operation is performed in continuous-wave mode with stability. Even though Nb offers such advantages, its QE is considerably low. Nb boasts stability and superconductivity, but its electron emission efficiency remains mediocre, making it relatively less suitable for applications requiring high QE. In such cases, techniques enhancing the photoemission yield are appropriate, and laser cleaning treatment is a primary method for rejuvenating the surface of Nb bulk-based photocathodes [56,77,78,79]. Relevant studies report the effectiveness of laser cleaning of Nb. Specifically, Q. Zhao and collaborators reported a significant increase in quantum efficiency (QE), from an initial value of 2.1 × 10^−7^ to 2.5 × 10^−5^, following laser cleaning of an Nb surface [77].

Devices built with a Nb thin film deposited on a Pb substrate have been studied to enhance QE. The good quality of Nb PLD films is demonstrated by their relevant adherence to the substrate, high hardness of 2.8 ± 0.3 GPa and relative high superconducting transition at 9.3 K [61]. The root-mean-square roughness value measured based on the AFM of the film deposited at room temperature is around 9 nm [80].

In Table 1, the relevant photoemissive properties of the most used bulk metallic photocathodes are reproduced and adapted from ref. [66]).

The results reported in Table 2 demonstrate the direct effect of laser cleaning on the QE value with the total number of laser pulses per site. The improvement in QE is largely due to the removal of contaminants and to the reduction in reflectivity of the photocathode surface [53,54]. Such precise and meticulous laser cleaning treatments are essential for enhancing the photoemission properties of metallic photocathodes.

## 5. Conclusions

This work reviews in situ laser cleaning treatments which yield a significant improvement in QE and enhance the operational stability of metallic photocathodes commonly used in RF and SRF gun applications. According to the present study, single-step cleaning with a few laser pulses per site is not an optimum method. Even in high-vacuum conditions, the presence of remaining reactive residues and their subsequent re-adsorption seriously degrades the quantum efficiency and overall performance.

The systematic investigation presented in this work concludes that laser cleaning acts not simply through oxide and hydride removing surface contaminants, but also via the further initiation of sequential chemical decontamination steps. These, in turn, minimize the concentration of reactive gas molecules, like H_2_ and H_2_O, in the vacuum vessel and consequently inhibit further adsorption and contamination. The present findings support the conclusion that only multi-steps laser cleaning can be an effective method for maintaining high photocathode QE over a long period of time, hence ensuring reliable photocathode performance under high-brightness conditions. Based on our conclusions, a practical recommendation to users is the application of step-wise, incremental laser treatment using extremely controlled laser irradiation at appropriate energy densities suited for each material. In this context, we have highlighted here the specific behavior of commonly used photocathode metals. As an example, the high reactivity of Y requires more stringent cleaning procedures to obtain a significant QE improvement. In comparison, the lower reactivity of Cu and Nb photocathodes makes them relatively resistant to chemical degradation, thereby requiring less intensive treatment. In the proposed approach, we can systematically remove contaminant layers without adversely affecting the surface of the photocathode. The slow regenerated purity of the metal surface is accompanied by secondary reactions which reduce contaminants in the vacuum vessel. Such proper environmental conditioning acts to progressively safeguard against re-contamination, thus maintaining the maximum photoemission yield.

In summary, metallic photocathodes remain an invaluable tool in RF and SRF gun technologies. Laser cleaning methods can provide optimal refurbishment of the cathodes by mitigating the effects of the chemical environment. Researchers and industrial users may follow the proposed guidelines to tailor laser cleaning procedures and maximize the quantum efficiency and overall performance of the photocathode, without compromising its surface integrity and long-term durability. The adaptability and versatility of laser cleaning make this method suitable for all types of metal photocathodes, thus constituting an enabling and critical technology for high-brightness electron beam machines.

## Figures and Tables

**Table 1:** Photoemissive properties of bulk metallic photocathodes.

	Cu	Mg	Y	Nb	Pb
Work function (eV)	4.6 [81]	3.6 [63]	3.1 [81]	4.3 [82]	4.2 [82]
QE (at 266 nm)/bulk	4.0 × 10^−5^ [74]	5 × 10^−4^ [65]	3.0 × 10^−4^ [45]	3.2 × 10^−6^ @248 nm [83]	7 × 10^−5^ [71]
Operational lifetime (yrs) [84]	Unlimited	~1	<1	Unlimited	>1
Compatibility with Cu RF gun	Very high	Medium	Low	-	-
Compatibility with Nb SCRF gun	-	-	-	Very high	High

**Table 2:** Effect of laser cleaning on photocathode QE.

Photocathode Material	QE Before Laser Cleaning	No. of Laser Cleaning Pulses per Site	QE After Laser Cleaning
Mg [27]	5.0 × 10^−4^	200	8.9 × 10^−4^
		300	1.1 × 10^−3^
		400	1.4 × 10^−3^
		500	1.8 × 10^−3^
Y [81]	~10^−5^	100	1.2 × 10^−4^
		400	2.8 × 10^−4^
		900	3.3 × 10^−4^
Pb *	3 × 10^−5^	>1000	8 × 10^−5^

* Nd:YAG laser; (4ω) λ = 266 nm; τ = 7 ns.

## Data Availability

No new data were created or analyzed in this study. Data sharing is not applicable to this article.

## Abbreviations

**Abbreviation**	**Definition**	
FEL	Free-electron laser	
ERL	Energy recovery linac	
UEM/D	Ultrafast electron microscopy and diffraction	
QE	Quantum efficiency	
RF	Radio frequency	
SRF	Superconducting radio frequency	
PLD	Pulsed laser deposition	
Nd:YAG	Neodymium-Doped Yttrium Aluminum Garnet	
SEM	Scanning Electron Microscopy	
UHV	Ultra-high vacuum	
HV	High vacuum	
**Symbol**		**Definition**
λ		Wavelength (nm)
τ		Laser pulse duration (s)
f		Repetition rate (Hz)
E		Laser energy density (J/cm^2^)
QE		Quantum efficiency
eV		Electron volt
amu		Atomic mass unit
µm		Micron
